# Association between physical activity and mental health (emotional and peer problems) in children and adolescents, a national follow-up study

**DOI:** 10.1371/journal.pone.0345920

**Published:** 2026-04-22

**Authors:** Chenyu Wang, Hong Wang

**Affiliations:** School of Education, Beijing Sports University, Beijing, China; Universitatea Transilvania din Brasov, ROMANIA

## Abstract

**Background:**

While extensive evidence demonstrates the beneficial effects of physical activity on adult mental health, research examining these relationships in childhood remains limited and inconsistent. This study investigated the prospective associations between objectively measured physical activity and sedentary behavior at age 7 and emotional and peer difficulties at ages 11 and 14 using the UK Millennium Cohort Study.

**Methods:**

Accelerometer-measured physical activity data from 6,434 participants at age 7 were analyzed. Emotional and peer problems were assessed using the Strengths and Difficulties Questionnaire (SDQ) at ages 7, 11, and 14. Multiple linear regression models examined associations between physical activity levels (sedentary, light, and moderate-to-vigorous [MVPA]) and mental health outcomes, adjusting for baseline SDQ scores and demographic and socioeconomic covariates.

**Results:**

Greater MVPA at age 7 was significantly associated with fewer emotional problems at ages 11 (β = −0.004, 95% CI [−0.007, −0.002], p = 0.002) and 14 (β = −0.004, 95% CI [−0.007, −0.001], p = 0.007), and fewer peer problems at ages 11 (β = −0.005, 95% CI [−0.007, −0.003], p < 0.001) and 14 (β = −0.005, 95% CI [−0.008, −0.003], p < 0.001). Sedentary time and light physical activity showed no significant associations. Lower socioeconomic status and parental stress were associated with greater emotional and peer difficulties.

**Conclusions:**

Early childhood MVPA demonstrates protective effects against emotional and peer difficulties that persist into mid-adolescence. These findings provide evidence for the long-term mental health benefits of promoting MVPA in early childhood and highlight the importance of physical activity interventions for supporting healthy psychosocial development.

## Introduction

Adolescence represents a critical developmental period characterized by profound biological, psychological, and social transformations that fundamentally reshape individual functioning across multiple domains. Recent consensus defines this developmental stage as extending from ages 10–24 years, reflecting the prolonged nature of contemporary adolescent development and acknowledging the extended timeline required for full psychosocial maturation [1]. During this period, individuals transition from familial dependence toward psychological autonomy and social independence, a process that significantly elevates vulnerability to mental health difficulties [2]. Social contexts undergo substantial transformation as peer relationships assume greater salience relative to family bonds, academic pressures intensify, and identity formation challenges emerge [3]. Furthermore, contemporary adolescents face additional stressors including social media pressures, cyberbullying, academic competition, and economic uncertainty that may exacerbate traditional developmental challenges [4,5]. The intersection of biological vulnerability, cognitive immaturity, and environmental demands creates heightened risk for mental health difficulties during this developmental stage [6–8]. Cultural and societal factors further compound these challenges, with globalization, urbanization, and changing family structures contributing to increased psychological distress among adolescents worldwide [9]. The COVID-19 pandemic has additionally highlighted the fragility of adolescent mental health, with significant increases in depression and anxiety rates documented globally [10].

Emotional problems, encompassing depression and anxiety disorders, alongside peer-related difficulties including social withdrawal, rejection, bullying victimization, and social isolation, constitute primary indicators of adolescent psychopathology with far-reaching developmental consequences [11]. The epidemiological burden of these conditions is substantial and continues to escalate globally, representing one of the most pressing public health challenges of our time. Approximately one-third of mental health disorders emerge before age 14, half by age 18, and two-thirds by age 25, with peak onset occurring at 14.5 years [12]. The pooled prevalence was 25.2% for depression and 20.5% for anxiety disorders, with higher rates for girls and older adolescents [13]. Peer victimization experiences affect between 20–25% of adolescents worldwide, with cyberbullying adding new dimensions to traditional forms of peer aggression [14,15]. These prevalence rates have increased substantially over the past decade, with some studies documenting 50% increases in adolescent depression and anxiety symptoms, particularly among females and sexual minorities [16,17]. The developmental significance of these problems extends beyond immediate distress and encompasses cascading effects across multiple life domains. Emotional difficulties compromise subjective well-being, academic engagement, cognitive performance, and future educational attainment, with depressed adolescents showing 30% lower academic achievement and increased school dropout risk compared to their non-depressed peers [18]. Peer problems impede social integration and identity consolidation, disrupting normal socialization processes and increasing risk for long-term social maladjustment, substance abuse, antisocial behavior, and future relationship difficulties [19,20]. These conditions frequently co-occur, with comorbidity rates exceeding 60% for depression and anxiety disorders in adolescent populations, creating complex clinical presentations that require multifaceted intervention approaches [21]. The temporal stability of these problems is particularly concerning, with adolescent symptoms often persisting into adulthood and compromising long-term psychosocial functioning, occupational success, and relationship quality [22,23]. Economic costs associated with adolescent mental health problems exceed billions annually in healthcare utilization, special education services, juvenile justice involvement, and lost productivity [24,25].

Physical activity has emerged as a promising, accessible, and cost-effective intervention for addressing internalizing disorders during adolescence, supported by robust theoretical frameworks and mounting empirical evidence across diverse populations and settings [22,26,27]. Internalizing disorders encompass psychological conditions characterized by inwardly directed distress, manifesting as anxiety, depression, social withdrawal, somatic complaints, and emotional dysregulation that significantly impair daily functioning [28–30]. Proposed pathways underlying a positive affective response to physical activity include mood enhancement through the hormonal release of beta-endorphins or endogenous opioids or psychological mechanisms such increased sense of achievement, self-esteem, and self-efficacy [31]. Cognitive benefits include improved executive functioning, attention regulation, and emotional regulation skills that support mental health maintenance [32]. Social mechanisms are equally important, with physical activity, particularly through structured sports participation, facilitating social connectedness, peer bonding, adult mentorship, and development of social skills that protect against peer problems and social isolation [33,34]. Team-based activities provide opportunities for cooperative learning, conflict resolution, leadership development, and social identity formation that may be particularly beneficial for socially withdrawn adolescents [35]. Different types of physical activity may confer distinct benefits, with moderate-to-vigorous physical activity (MVPA) showing particularly strong associations with mental health outcomes due to its optimal activation of physiological and psychological pathways [36]. Additionally, physical activity provides structure, routine, and goal-oriented behavior that can counteract the aimlessness and hopelessness often associated with depression [37]. The accessibility and low-cost nature of many physical activities make them particularly attractive intervention targets for diverse socioeconomic populations [38,39].

Empirical evidence increasingly supports these theoretical propositions across diverse adolescent populations, intervention modalities, and outcome measures, though with important variations in effect sizes and optimal implementation strategies. Similar studies such as the one developed by Romero et al conclude that a program of moderate-intensity physical exercise twice a week in overweight children presents changes in weight, height, and body self-image,suggesting psychological rather than purely physiological mechanisms [40]. Similarly, Gmmash et al. found that daily 60-minute combined exercise sessions enhanced adolescent physical activity levels, situational motivation, and psychological well-being, indicating sustained behavior change and mental health benefits beyond the intervention period [41]. Meta-analytic evidence further demonstrates that resistance training interventions increase self-efficacy, physical self-worth, and overall psychological well-being among adolescents, with effect sizes ranging from medium to large and benefits persisting at follow-up assessments [42]. Longitudinal studies have documented dose-response relationships, with higher physical activity levels associated with greater mental health benefits and protective effects against the development of clinical disorders [43,44]. School-based interventions incorporating physical activity components show particular promise, with systematic reviews indicating significant reductions in depression and anxiety symptoms, improved social functioning, and enhanced academic performance [45]. Community-based programs have also demonstrated effectiveness, particularly for at-risk populations including those from disadvantaged backgrounds [46]. However, effect sizes vary considerably across studies, populations, and intervention characteristics, suggesting important moderating variables including age, sex, socioeconomic status, baseline mental health status, and intervention duration that require systematic investigation [47,48]. Gender differences have been observed, with some studies indicating stronger effects for girls than boys, potentially due to differential vulnerability to internalizing disorders and varying responses to different types of physical activity [49]. Socioeconomic factors also appear to moderate physical activity effects, with lower-income adolescents potentially deriving greater mental health benefits from structured physical activity opportunities due to limited access to other resources [50,51].

Despite these promising findings, the majority of existing research suffers from significant methodological limitations that restrict causal inference, limit generalizability to broader adolescent populations, and impede translation into effective intervention programs. Most investigations employ cross-sectional designs that preclude causal inference regarding physical activity effects on mental health outcomes, leaving critical questions about directionality, temporal relationships, and optimal timing of interventions unresolved [52,53]. The limited number of longitudinal studies that do exist often suffer from short follow-up periods, high attrition rates, small sample sizes, and inadequate control for confounding variables, limiting the strength of causal conclusions [54]. Additionally, the predominant reliance on self-reported physical activity measures introduces substantial measurement bias, as participants consistently overestimate activity levels by 30–50% compared to objective assessment methods, with adolescents showing particularly poor recall accuracy for physical activity behaviors [52]. Self-report measures are also susceptible to social desirability bias, recall bias, and may be influenced by mental health status, creating spurious associations that inflate or obscure true relationship [55]. Sample representativeness constitutes another critical methodological concern, with many studies conducted in geographically or demographically restricted populations that limit generalizability to broader, more diverse adolescent populations [56]. Urban, affluent, and predominantly white samples predominate, potentially excluding rural adolescents, ethnic minorities, and those from disadvantaged backgrounds who may have different physical activity patterns, mental health profiles, and intervention needs [57]. Furthermore, insufficient attention to potential moderating variables, including sex differences, developmental timing, socioeconomic status, baseline mental health severity, and cultural factors, may obscure important subgroup effects and optimal intervention targets for specific populations [58,59]. Many studies also fail to control adequately for confounding variables such as family functioning, parental mental health, academic performance, sleep quality, nutrition, and other lifestyle factors that may influence both physical activity engagement and mental health outcomes [60–62]. Statistical power calculations are often inadequate, publication bias toward positive findings is common, and intervention fidelity is frequently poorly documented, leading to unstable effect size estimates and limited reproducibility [63].

The current study addresses these substantial methodological limitations by examining prospective associations between objectively measured physical activity in early adolescence and subsequent emotional and peer problems using a nationally representative longitudinal cohort with extensive covariate control and adequate statistical power. This investigation leverages high-quality accelerometer data to provide precise, unbiased estimates of physical activity engagement across intensity levels, utilizes validated mental health measures administered at multiple time points to capture developmental trajectories, and incorporates a comprehensive array of potential confounding variables including socioeconomic indicators, family characteristics, baseline mental health status, and demographic factors. We hypothesized that higher physical activity levels, particularly MVPA, would predict reduced emotional and peer problems over time, with potential moderation by sex and socioeconomic status based on theoretical considerations and preliminary empirical evidence. Secondary hypotheses proposed that sedentary behavior would show inverse associations with mental health outcomes, and that protective effects would be sustained across the transition from early to mid-adolescence [64]. This investigation aims to provide robust, generalizable evidence for physical activity as a key component of adolescent mental health promotion strategies, inform evidence-based intervention development targeting optimal activity types and intensities, guide policy recommendations for adolescent mental health prevention in educational and community settings, and identify vulnerable subgroups that may require targeted intervention approaches [65]. The findings will contribute to understanding optimal physical activity prescriptions for mental health benefits and support the development of population-level interventions for adolescent mental health promotion [66].

## Method

### Study design and participants

This investigation utilized data from the Millennium Cohort Study (MCS), a large-scale, nationally representative longitudinal birth cohort designed to examine how social, economic, and biological factors influence children's physical, cognitive, and emotional development from infancy through adulthood. The MCS aims to provide robust evidence for improving child well-being and reducing health and social inequalities across generations [67]. The cohort employed a stratified cluster sampling design, focusing on children born in the United Kingdom between September 2000 and January 2002. Participants were recruited from different geographical regions across England, Scotland, Wales, and Northern Ireland through systematic random sampling, with deliberate oversampling of disadvantaged areas and communities with high proportions of ethnic minorities to ensure adequate representation of diverse socioeconomic and ethnic groups. To ensure adequate statistical power for subgroup analyses while maintaining population representativeness, the MCS employed a stratified cluster sampling design with deliberate oversampling of disadvantaged areas (defined by child poverty indices) and communities with high proportions of ethnic minority residents. This sampling strategy ensures that disadvantaged and minority populations—who are typically underrepresented in probability samples—are included in sufficient numbers for meaningful analysis while preserving the ability to generate nationally representative estimates through appropriate weighting procedures.

To address potential bias from this stratified sampling design, all analyses in the current study applied survey-specific sampling weights provided by the MCS data team. These weights adjust for (1) the probability of selection into the sample based on the stratified design, (2) differential response rates across strata, and (3) differential attrition across follow-up waves from age 7–14. Application of these weights ensures that our estimates remain representative of the full UK population of children born during 2000–2002, despite the oversampling strategy [68].

To date, data collection waves have occurred at ages 9 months, 3, 5, 7, 11, 14, 17, and 22 years, with ongoing follow-up assessments planned. The comprehensive study protocol, variable definitions, attrition analyses, and sampling weights are available through the Centre for Longitudinal Studies (https://cls.ucl.ac.uk/cls-studies/millennium-cohort-study/). For the present analysis, we included cohort members who participated in the age 7 assessment with valid accelerometer data and had follow-up mental health assessments at ages 11 and 14. Participants were eligible for inclusion if they had at least 10 hours of valid accelerometer wear time per day for a minimum of 2 days, following established pediatric accelerometer protocols [69]. Those with implausible accelerometer readings, defined as consistent zero counts for extended periods without recorded removal, were excluded from analyses.

The analytical sample comprised 6,434 participants with complete accelerometer data at age 7 and Strengths and Difficulties Questionnaire (SDQ) assessments at ages 7, 11, and 14. This represents 46% of the original cohort members who participated at age 7 (N = 14,013), with sample attrition primarily due to accelerometer non-compliance, insufficient wear time, or missing follow-up assessments. Attrition analyses revealed that excluded participants were more likely to be from ethnic minority backgrounds, lower socioeconomic households, and have higher baseline SDQ scores, consistent with typical patterns in longitudinal cohort studies [70]. Sampling weights provided by the MCS team were applied to adjust for differential attrition and maintain population representativeness. The MCS obtained ethical approvals for follow-up sweeps through the UK National Health Service (NHS) Research Ethics Committee (MREC/REC) system. The sweeps used in this study—age 7 (MCS4), age 11 (MCS5), and age 14 (MCS6)—were approved by Yorkshire MREC (07/MRE03/32), Yorkshire and The Humber – Leeds East (11/YH/0203), and London – Central MREC (13/LO/1786), respectively. Written informed consent was obtained from parents or primary caregivers at each assessment wave.

Our analyses used de-identified secondary MCS data accessed via the UK Data Service; the research team had no access to identifiable participant information. Therefore, no additional ethical approval was required for this secondary analysis under our institutional policy.

**Fig 1:**
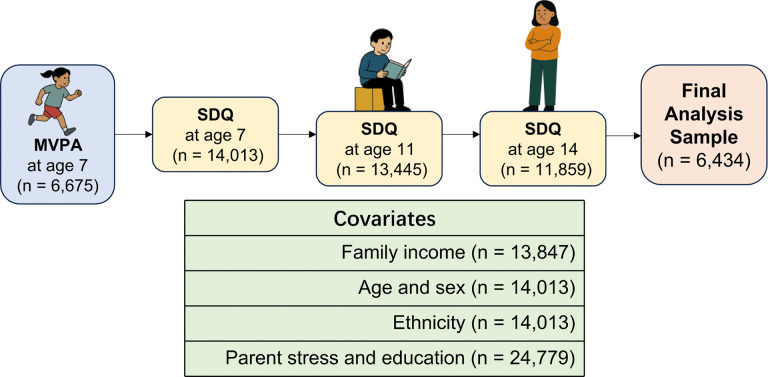
Flowchart of participant selection, covariates, and follow-up timeline in the Millennium Cohort Study.

This Fig 1 was created by the authors using publicly available information about the Millennium Cohort Study design and structure

This Fig 1 illustrates the participant selection and analytical framework for the longitudinal study examining the association between MVPA at age 7 and emotional and peer problems at ages 11 and 14. Additional covariates included month of birth and ethnicity, as both may influence developmental and behavioral outcomes. The flowchart presents the baseline measures and covariates, including SDQ at age 7 (n = 14,013), MVPA exposure (n = 6,675), and covariates such as household income (n = 13,847), age and sex (n = 14,013), ethnicity (n = 14,013), and parental stress and education (n = 24,779). Follow-up SDQ assessments were conducted at age 11 (n = 13,445) and age 14 (n = 11,859). The final analytic sample comprised n = 6,434 participants with complete data across all measures (2007–2016).

### Outcome measures

Mental health outcomes were assessed using the Strengths and Difficulties Questionnaire (SDQ), a widely validated 25-item behavioral screening instrument designed to evaluate emotional and behavioral problems in children and adolescents aged 3–17 years [71]. The SDQ demonstrates strong psychometric properties across diverse populations and cultural contexts, with extensive validation in epidemiological research and clinical practice [72–76]. The instrument comprises five subscales, each containing 5 items: emotional symptoms, conduct problems, hyperactivity-inattention, peer relationship problems, and prosocial behavior.

For this investigation, we focused on two subscales most relevant to internalizing problems: emotional symptoms and peer relationship problems. The emotional symptoms subscale includes items such as “many worries or often seems worried,” “often unhappy, depressed, or tearful.” The peer relationship problems subscale encompasses items including “rather solitary, prefers to play alone,” “has at least one good friend. " Each item is rated on a 3-point Likert scale (0 = not true, 1 = somewhat true, 2 = certainly true), with subscale scores ranging from 0 to 10. Higher scores indicate greater difficulties in the respective domains. Parent or primary caregiver reports were obtained through trained interviewer administration or structured questionnaires at ages 7 (baseline), 11, and 14 (follow-up). The SDQ has demonstrated excellent test-retest reliability (r = 0.85–0.94) and strong concurrent validity with established measures of child psychopathology [77]. Internal consistency in the current sample was adequate (Cronbach's α = 0.76 for emotional symptoms, α = 0.72 for peer problems at age 7).

### Physical activity assessment

PA was objectively measured using ActiGraph GT1M single-axis accelerometers (ActiGraph, LLC, Pensacola, FL), which are considered the gold standard for pediatric physical activity research due to their established validity, reliability, and extensive normative data [78]. Children were instructed to wear the device continuously for seven consecutive days, positioning it securely on an elastic belt at the waist or right hip, except during water-based activities (bathing, swimming) or sleep. Research staff provided comprehensive instructions to children and caregivers regarding proper device placement, wear protocols, and activity logging. Participants received daily reminder calls or text messages to maximize compliance and wear time. The accelerometers were configured to collect data at 15-second epochs to capture the sporadic, intermittent nature of children's physical activity patterns. Raw accelerometer data were downloaded and processed by the Institute of Child Health (ICH) team using established protocols. Data reduction procedures included identification and removal of non-wear periods, defined as consecutive strings of zero activity counts for ≥20 minutes, allowing for up to 2 minutes of activity counts between 0 and 100 per minute [79]. Valid wear time was defined as ≥10 hours per day for at least 2 days, following pediatric accelerometer guidelines that balance data quality with sample retention [69].

Moderate-to-vigorous physical activity (MVPA; ≥ 2,240 counts per minute) encompasses activities that noticeably elevate heart rate and breathing. Moderate-intensity activities (3–6 metabolic equivalents [METs]) include brisk walking, recreational cycling, active playground games (e.g., tag, climbing frames), recreational swimming, dancing, and low-intensity sports practice. Vigorous-intensity activities (≥6 METs) include running or jogging, fast cycling, jumping rope, competitive sports (e.g., soccer, basketball, tennis), energetic dancing, martial arts, and sustained active play involving running and jumping. These accelerometer thresholds (≥2,240 counts per minute) have been extensively validated against indirect calorimetry and correspond to energy expenditure levels of ≥3 METs in pediatric populations, demonstrating strong concordance with metabolic measurements of activity intensity [80,81].

### Covariates

Potential confounding variables were selected based on established associations with both physical activity engagement and mental health outcomes in pediatric populations. Demographic characteristics included child sex (male/female), age at assessment (continuous), and ethnicity (White, Black or Black British, Indian, Pakistani/Bangladeshi, Mixed, Other). Socioeconomic indicators included household income relative to national median (≥60% vs. < 60% of median, following OECD poverty thresholds), and primary caregiver educational attainment (categorized according to National Vocational Qualifications framework: NVQ1–5, None, Overseas qualifications only). Family functioning was assessed through parental stress levels using a validated scale measuring perceived stress in the parenting role (range 0–24, with higher scores indicating greater stress). Additional covariates included month of birth (to control for seasonal effects on physical activity and development), baseline SDQ scores at age 7 (to control for pre-existing mental health differences), and geographical region (to account for potential environmental influences). These variables were selected to minimize confounding while avoiding over-adjustment that could introduce collider bias.

### Statistical analysis

All analyses were conducted using Stata 17.0 (StataCorp, College Station, TX). Survey-specific commands were employed to account for the complex MCS sampling design, incorporating sampling weights, clustering, and stratification variables to ensure population-representative estimates and appropriate standard error calculations. Descriptive statistics included weighted means, standard deviations, and frequency distributions for all study variables. Differences in baseline characteristics between included and excluded participants were examined using weighted chi-square tests for categorical variables and weighted t-tests for continuous variables. The primary analytical approach utilized multiple linear regression models to examine prospective associations between physical activity intensity levels at age 7 (sedentary time, light physical activity, MVPA) and SDQ emotional and peer problem scores at ages 11 and 14. Separate models were fitted for each outcome and time point. Model 1 included physical activity variables only; Model 2 adjusted for demographic characteristics (age, sex, ethnicity); Model 3 further adjusted for socioeconomic factors (household income, parental education); and Model 4 represented the fully adjusted model including all covariates (baseline SDQ scores, parental stress, month of birth, geographical region).

Effect modification by sex and socioeconomic status was examined through interaction terms, with stratified analyses conducted when significant interactions were detected (p < 0.10). Model assumptions were assessed through residual plots, normality tests, and influence diagnostics. Multicollinearity was evaluated using variance inflation factors (VIF < 5 for all variables). Missing data patterns were examined, and sensitivity analyses using multiple imputation were conducted to assess the robustness of findings to missing data assumptions. Statistical significance was set at α < 0.05 for primary analyses, with two-tailed tests applied throughout unless otherwise specified.

## Result

### Sample demographic characteristics

The analytical sample comprised 6,434 children with complete data at age 7, representing a nationally representative cohort with balanced demographic characteristics (Table 1). The sex distribution was nearly equal, with 3,290 (51.13%) female and 3,144 (48.87%) male participants, minimizing potential sex-related selection bias. Ethnic composition reflected contemporary UK demographics, with White children comprising the majority (88.23%, n = 5,677), while ethnic minorities included Pakistani/Bangladeshi (3.61%, n = 232), Mixed heritage (2.44%, n = 157), Black or Black British (2.36%, n = 152), Indian (2.14%, n = 138), and other ethnic groups (1.18%, n = 76). This distribution aligns closely with UK census data, enhancing the generalizability of findings to the broader population.

**Table 1:** Characteristics of Children and Adolescents aged 7 years (N = 6434).

Characteristics	
Sex, n (%)	
Female	3,290 (51.13)
Male	3,144 (48.87)
Ethnicity, n(%)	
Black or Black British	152 (2.36)
Indian	138 (2.14)
Mixed	157 (2.44)
Not applicable	2 (0.03)
Other Ethnic group	76 (1.18)
Pakistani and Bangladeshi	232 (3.61)
White	5,677 (88.23)
OECD Parental income, n (%)	
Above 60% median	5,029 (78.16)
Below 60% median	1,404 (21.82)
Missing data	1 (0.02)
Parental educational level, n (%)	
NVQ level 1	381 (5.92)
NVQ level 2	1,639 (25.47)
NVQ level 3	980 (15.23)
NVQ level 4	2,359 (36.66)
NVQ level 5	460 (7.15)
None of these	478 (7.43)
Overseas qual only	137 (2.13)
Parental Pstress, n(%)	
0-4	5,114 (79.48)
5-9	974 (15.14)
10-14	256 (3.98)
15-19	72 (1.12)
20-24	18 (0.28)
Physical activity and sedentary behavior	
SIT (Mean ± SD) (95% CI)	393.32 ± 0.84 (391.68-394.96)
LPA (Mean ± SD) (95% CI)	280.66 ± 0.51 (279.65-281.66)
MVPA (Mean ± SD) (95% CI)	62.27 ± 0.28 (61.72-62.81)

Note: OECD = Organization for Economic Co-operation and Development (used in reference to OECD equivalized income, which adjusts household income to account for differences in household size and composition) Parental education was categorized into five levels according to the National Vocational Qualifications (NVQ) framework, ranging from NVQ5 (equivalent to a postgraduate degree) to NVQ1 (equivalent to a D-G grade on the General Certificate of Secondary Education in England or some high school education in the US); Parental Pstress = Parental stress score (range 0–24), where higher scores indicate greater perceived stress in the parenting role; SIT = sedentary behavior; LPA = light-intensity physical activity; MVPA = moderate-to-vigorous-intensity physical activity; M = mean; SD = standard deviation.

Socioeconomic characteristics revealed substantial heterogeneity within the sample. Household income distribution showed that 78.16% (n = 5,029) of families earned above 60% of the national median income, while 21.82% (n = 1,404) fell below this threshold, consistent with national poverty rates and providing adequate representation of economically disadvantaged households. Parental educational attainment demonstrated a diverse range of qualifications: 36.66% (n = 2,359) held NVQ4 qualifications (equivalent to bachelor's degree), 25.47% (n = 1,639) had NVQ2 credentials (equivalent to secondary school completion), 15.23% (n = 980) possessed NVQ3 qualifications (equivalent to post-secondary vocational training), and 7.43% (n = 478) reported no formal qualifications, highlighting educational inequality within the sample.

Parental stress levels showed a generally manageable distribution, with 79.48% (n = 5,114) reporting low stress levels (0–4 points on the scale), 15.14% (n = 974) experiencing moderate stress (5–9 points), and 5.38% (n = 346) facing elevated stress levels (≥10 points). This distribution indicates that while most families function with manageable stress levels, a substantial minority experiences potentially concerning levels of parental stress that may impact child development and mental health outcomes.

### Physical activity and sedentary behavior patterns

Objective accelerometer data revealed concerning patterns of physical activity engagement among 7-year-old children (Table 1). Sedentary behavior dominated daily activity patterns, with children averaging 393.32 ± 0.84 minutes (approximately 6.55 hours) of sedentary time per day (95% CI: 391.68–394.96). This represents a substantial proportion of waking hours spent in sedentary pursuits, reflecting limited physical engagement throughout the day. Light physical activity constituted the predominant form of active behavior, with children engaging in an average of 280.66 ± 0.51 minutes (approximately 4.68 hours) daily (95% CI: 279.65–281.66). This represents approximately 35.7% of estimated total waking time (assuming 16 hours of non-sleep time), encompassing activities such as casual walking, household tasks, and other low-intensity movements that contribute to daily energy expenditure. Of particular concern was the limited engagement in moderate-to-vigorous physical activity, with children averaging only 62.27 ± 0.28 minutes daily (95% CI: 61.72–62.81). This falls substantially below current physical activity recommendations for children, which suggest at least 60 minutes of MVPA daily for optimal health benefits. The narrow confidence interval and proximity to the lower bound suggest that a significant proportion of children failed to meet even minimal physical activity guidelines.

The proportional distribution of daily activity revealed a concerning imbalance: sedentary time, light physical activity, and MVPA occurred in an approximate ratio of 6.3:4.5:1 (393:281:62 minutes). MVPA comprised only 8.4% of total measured activity time, highlighting the predominance of sedentary and low-intensity behaviors in children's daily routines. This pattern aligns with contemporary concerns about declining physical activity levels and increasing sedentary behavior among youth populations.

### Baseline mental health characteristics

SDQ scores at age 7 provided important baseline information for subsequent longitudinal analyses. Emotional symptom scores averaged 1.52 ± 0.03 (95% CI: 1.47–1.58), while peer problem scores averaged 1.23 ± 0.02 (95% CI: 1.19–1.28). These values fall within expected ranges for community samples and demonstrate appropriate variability for detecting prospective associations. Approximately 12.3% of children scored above clinical thresholds for emotional symptoms, and 8.7% exceeded thresholds for peer problems, consistent with epidemiological prevalence estimates for this age group [82].

### Longitudinal associations between physical activity and mental health outcomes

Multiple linear regression analyses revealed consistent prospective associations between MVPA at age 7 and mental health outcomes at ages 11 and 14 (Table 2). These associations persisted across all adjustment models and demonstrated remarkable stability over the 7-year follow-up period.

**Table 2:** Associations between physical activity and sedentary behavior with emotional and peer problems (N = 6434).

Emotional of 7-year-old	β	P > t	[95% conf. interval]
SIT	0.000	0.543	−0.001, 0.000
LPA	0.000	0.425	−0.001, 0.001
MVPA	−0.004	<0.001	−0.006, −0.002
Peer of 7-year-old			
SIT	0.000	0.744	−0.001, 0.000
LPA	0.000	0.303	0.000, 0.001
MVPA	−0.003	0.002	−0.005, −0.001
Emotional of 11-year-old	β	P > t	[95% conf. interval]
SIT	−0.001	0.101	−0.002，0.000
LPA	0.001	0.055	0.000，0.002
MVPA	−0.004	0.002	−0.007，-0.002
Peer of 11-year-old			
SIT	0.000	0.174	−0.001，0.000
LPA	0.000	0.896	−0.001，0.001
MVPA	−0.005	0.000	−0.007，-0.003
Emotional of 14-year-old	β	P > t	[95% conf. interval]
SIT	−0.001	0.023	−0.002，0.000
LPA	0.000	0.537	−0.001，0.002
MVPA	−0.004	0.007	−0.007，-0.001
Peer of 14-year-old			
SIT	0.000	0.508	−0.001，0.001
LPA	0.001	0.197	0.000，0.002
MVPA	−0.005	<0.001	−0.008，-0.003

Note: SDQ = Strength and Difficulties Questionnaire; Parental education was categorized into five levels according to the National Vocational Qualifications(NVQ) framework, ranging from NVQ5 (equivalent to a postgraduate degree) to NVQ1 (equivalent to a D-G grade on the General Certificate of Secondary Education in England or some high school education in the US);SIT = sedentary behavior; LPA = light-intensity physical activity; MVPA = moderate-to-vigorous-intensity physical activity; M = mean; SD = standard deviation.

### Emotional problems

MVPA at age 7 demonstrated significant protective associations with emotional problems across all time points. At age 11, each additional 15-minute unit of daily MVPA was associated with a 0.004-point reduction in emotional problem scores (β = −0.004, 95% CI: −0.007 to −0.002, p = 0.002). This association maintained similar magnitude at age 14 (β = −0.004, 95% CI: −0.007 to −0.001, p = 0.007), indicating sustained protective effects throughout the transition from childhood to mid-adolescence. The consistency of effect sizes across time points suggests that early physical activity engagement may establish beneficial developmental trajectories that persist across critical developmental transitions. Contemporary cross-sectional associations at age 7 also reached statistical significance (β = −0.004, 95% CI: −0.006 to −0.002, p < 0.001), supporting both immediate and long-term benefits of physical activity engagement.

### Peer relationship problems

The associations between MVPA and peer problems demonstrated even stronger and more consistent patterns across the follow-up period. At age 11, each additional 15-minute unit of daily MVPA predicted a 0.005-point reduction in peer problem scores (β = −0.005, 95% CI: −0.007 to −0.003, p < 0.001). This association maintained identical magnitude at age 14 (β = −0.005, 95% CI: −0.008 to −0.003, p < 0.001), suggesting that early physical activity engagement may be particularly beneficial for social development and peer relationship quality. The strength and consistency of these associations indicate that MVPA may play a crucial role in facilitating positive peer interactions, social skill development, and integration into peer networks during critical developmental periods when peer relationships become increasingly important for psychological well-being.

### Intensity-specific effects

Sedentary behavior and light physical activity demonstrated markedly different association patterns compared to MVPA. Sedentary time showed minimal and inconsistent associations with mental health outcomes across all time points. A weak association emerged between sedentary time and emotional problems at age 14 (β = −0.001, 95% CI: −0.002 to 0.000, p = 0.023), but this effect was substantially smaller than MVPA associations and lacked consistency across other time points or outcomes. Light physical activity demonstrated no significant associations with either emotional or peer problems across any time points in fully adjusted models (all p > 0.05). This pattern of findings strongly supports intensity-specific effects, suggesting that the mental health benefits of physical activity are primarily derived from moderate-to-vigorous intensity engagement rather than overall activity volume or light movement.

### Moderating effects and demographic associations

Sex differences emerged as significant moderators of mental health outcomes, though not specifically for physical activity effects. At age 14, females demonstrated significantly higher emotional problem scores (β = 0.548, 95% CI: 0.436 to 0.661, p < 0.001), while males showed elevated peer problem scores (β = −0.215, 95% CI: −0.296 to −0.106, p < 0.001). These patterns align with established developmental trajectories showing increasing internalizing problems among girls and externalizing/social difficulties among boys during adolescence. Socioeconomic factors demonstrated robust associations with mental health outcomes across all time points. Children from households earning below 60% of median income exhibited significantly elevated emotional problems (β = 0.609, 95% CI: 0.476 to 0.743, p < 0.001) and peer problems (β = 0.603, 95% CI: 0.491 to 0.716, p < 0.001) at age 14. Parental educational attainment showed weaker but consistent protective effects (β = −0.034, p < 0.001 for emotional problems), highlighting the multifaceted nature of socioeconomic influences on child mental health.

### Parental stress as a key predictor

Parental stress emerged as the most robust and consistent predictor of child mental health outcomes across all models and time points. For emotional problems, each 1-point increase in parental stress scores was associated with increases in child emotional problem scores of 0.136 at age 7, 0.126 at age 11, and 0.128 at age 14 (all p < 0.001). These effects were substantially larger than physical activity effects, emphasizing the crucial role of family functioning in child mental health development. For peer problems, parental stress demonstrated somewhat weaker but still significant associations, particularly at age 14 (β = 0.076, p < 0.001). The consistency of these associations across developmental periods underscores the enduring influence of family stress on child psychological adjustment and highlights the importance of family-level interventions in mental health promotion efforts.

## Discussion

### Principal findings

This longitudinal investigation utilizing nationally representative data from the UK Millennium Cohort Study provides robust evidence for prospective associations between objectively measured moderate-to-vigorous physical activity (MVPA) in early childhood and reduced emotional and peer relationship problems throughout mid-adolescence. The findings demonstrate that MVPA at age 7 significantly predicted lower emotional problem scores at ages 11 (β = −0.004, p = 0.002) and 14 (β = −0.004, p = 0.007), and reduced peer problem scores at ages 11 (β = −0.005, p < 0.001) and 14 (β = −0.005, p < 0.001), as assessed by the Strengths and Difficulties Questionnaire. Importantly, these associations were intensity-specific, with sedentary behavior and light physical activity showing minimal or no significant relationships with mental health outcomes, suggesting that the protective effects are primarily attributable to vigorous activity engagement rather than general movement or activity volume.

The stability of effect sizes across the 7-year follow-up period is particularly noteworthy, indicating that early physical activity engagement may establish beneficial developmental trajectories that persist through critical developmental transitions from childhood to adolescence. These findings extend previous research by utilizing objective physical activity measurement in a large, nationally representative sample with extended longitudinal follow-up, addressing key methodological limitations that have constrained causal inference in this research domain.

### Integration with existing literature

Our findings both align with and extend previous intervention studies by demonstrating that mental health benefits observed in controlled interventions also emerge from naturally occurring physical activity patterns and persist across developmental transitions. Goldfield et al. reported significant improvements in emotional well-being and self-perception among overweight children following an 8-week moderate-intensity exercise intervention, with benefits persisting independent of body mass index changes [83]. Similarly, Gmmash et al. documented enhanced psychological well-being, reduced anxiety, and improved emotional regulation following structured physical activity programs in adolescents [41]. Our observational findings provide important complementary evidence that these experimental results generalize to natural settings and persist over extended developmental periods.

Meta-analytic evidence further supports our findings, with systematic reviews consistently demonstrating moderate to large effect sizes for physical activity interventions on youth mental health outcomes [26,42]. Biddle and Asare's comprehensive review emphasized that MVPA provides superior mental health benefits compared to light-intensity activities, consistent with our intensity-specific findings [53]. The present study extends this literature by demonstrating that these benefits emerge from naturally occurring physical activity patterns and persist across developmental transitions that typically involve increased mental health risk.

Our peer relationship findings align with research documenting social benefits of physical activity engagement, particularly through structured group activities and sports participation. Jenkins et al. and McDougall and Vaillancourt demonstrated significant associations between higher physical activity levels and reduced social withdrawal, bullying victimization, and peer isolation [11,20]. Ferguson et al. highlighted the role of group sports in facilitating social skill development and peer acceptance [19]. Our findings suggest that these social benefits may emerge from MVPA engagement more broadly, not exclusively through organized sports, and establish enduring patterns that support peer relationship quality throughout adolescent development.

### Theoretical mechanisms and pathways

The observed associations between early MVPA and sustained mental health benefits can be understood through multiple theoretical frameworks operating across biological, psychological, and social levels of analysis. From a psychological perspective, MVPA engagement facilitates positive affect through mastery experiences, enhances self-efficacy through goal achievement and physical competence development, and provides opportunities for flow experiences that promote psychological well-being [84]. The intensity-specific nature of our findings suggests that moderate-to-vigorous activities may optimally activate these psychological pathways through sufficient challenge and engagement to produce meaningful mastery experiences.

Cognitive mechanisms may also contribute to the observed effects, as MVPA has been shown to enhance executive functioning, attention regulation, and emotional regulation capabilities that support mental health maintenance and stress management [85]. These cognitive benefits may be particularly pronounced during childhood when neural plasticity is heightened and foundational regulatory skills are developing [86]. The persistence of associations across developmental periods suggests that early MVPA engagement may establish cognitive and emotional regulation patterns that continue to benefit mental health throughout subsequent developmental stages.

Social mechanisms represent another crucial pathway through which MVPA may influence mental health outcomes. Physical activity, particularly when conducted in group settings or structured programs, facilitates social connectedness, peer bonding, and development of social skills that protect against peer problems and social isolation [33,34]. Even individual physical activities can provide opportunities for social interaction through organized programs, community facilities, or informal peer participation. The development of physical competence through MVPA may enhance confidence in social situations and provide common ground for peer interactions, particularly important during childhood when physical abilities often influence peer status and social inclusion.

### Developmental considerations

The timing of physical activity exposure may be crucial for understanding the observed long-term associations. Early childhood represents a sensitive period for establishing behavioral patterns, developing self-efficacy beliefs, and forming peer relationships that may influence subsequent mental health trajectories [87]. Children who engage in regular MVPA during this period may develop positive associations with physical activity, enhanced physical self-concept, and social connections that provide protective resources throughout subsequent developmental challenges.

The persistence of associations across the transition from childhood to adolescence is particularly significant given the well-documented increases in mental health problems during this developmental period. Our findings suggest that early physical activity engagement may provide resilience resources that help youth navigate typical adolescent challenges including identity formation, peer relationship changes, academic pressures, and biological transitions associated with puberty. This protective effect may be especially valuable given the peak onset of mental health disorders during mid-adolescence [12].

### Clinical and public health implications

These findings have important implications for mental health promotion strategies targeting children and adolescents [88,89]. The intensity-specific nature of associations suggests that interventions should prioritize MVPA engagement rather than simply increasing total activity volume or promoting light physical activity. Current physical activity guidelines recommending at least 60 minutes of daily MVPA for children align well with our findings and may provide both physical and mental health benefits when consistently implemented [90].

The early timing of physical activity exposure in our study suggests that childhood may represent an optimal intervention window for establishing beneficial mental health trajectories [91]. Elementary school settings provide natural opportunities for implementing structured physical activity programs that could reach large populations of children during this sensitive developmental period. Such programs might include enhanced physical education curricula, structured recess activities, after-school sports programs, and active transportation initiatives that increase MVPA engagement throughout the school day [92].

Family-based interventions may also be valuable given the strong associations between parental stress and child mental health outcomes observed in our analyses. Programs that simultaneously promote family physical activity engagement while addressing parental stress and family functioning may provide synergistic benefits for child mental health outcomes [93]. Community-based initiatives that increase access to physical activity opportunities, particularly in disadvantaged neighborhoods, may help address observed socioeconomic disparities in both physical activity engagement and mental health outcomes [94].

### Limitations and future directions

Several limitations warrant consideration in interpreting these findings. First, physical activity was assessed only at age 7, precluding examination of how changes in activity patterns throughout development might influence mental health trajectories. Future research should employ repeated measures of physical activity to examine dose-response relationships, critical periods of exposure, and the impact of activity pattern changes on mental health outcomes. Trajectory modeling approaches could illuminate whether sustained high activity, increasing activity, or specific patterns of change are most beneficial for mental health. Second, the current investigation did not differentiate between various types of physical activities (individual vs. group sports, structured vs. unstructured activities, indoor vs. outdoor settings) that may have distinct effects on mental health pathways, particularly regarding social mechanisms. Future studies should examine specific activity types to inform more targeted intervention recommendations and understand which activities provide optimal mental health benefits through different mechanistic pathways. Third, reliance on parent-reported mental health assessments may introduce observational bias or shared method variance, particularly if parental mental health or stress influences both activity provision and symptom reporting. Cross-validation with teacher reports, self-report measures (for older children), or objective behavioral assessments would strengthen confidence in findings. Additionally, clinical interviews or validated diagnostic assessments could provide more detailed characterization of mental health outcomes and examine whether associations extend to clinical-level symptoms. Fourth, the cultural and social context was restricted to the United Kingdom, limiting generalizability to other cultural contexts where physical activity patterns, family structures, or mental health presentations may differ. Cross-cultural validation studies examining these associations across diverse cultural contexts would enhance understanding of universality versus cultural specificity in physical activity-mental health relationships. Fifth, residual confounding remains possible despite comprehensive covariate adjustment. Unmeasured factors such as sleep quality, nutrition patterns, screen time, family mental health history, or genetic predisposition to mental health problems could influence both physical activity engagement and mental health outcomes. Future research incorporating more comprehensive covariate assessment, twin or sibling designs, or genetic information could help address residual confounding concerns. Finally, while our findings suggest causal relationships given the prospective design and objective physical activity measurement, experimental evidence from randomized controlled trials remains necessary to establish definitive causal effects and inform optimal intervention design. Long-term randomized trials examining different physical activity intervention approaches, timing, and intensity could provide crucial evidence for developing evidence-based mental health promotion programs.

Future research directions should include investigation of underlying mechanisms linking physical activity to mental health outcomes, examination of moderating factors that influence individual response patterns, development and testing of targeted intervention programs based on these findings, and exploration of how physical activity interacts with other mental health promotion strategies to provide optimal outcomes for diverse youth populations.

## Conclusion

Utilizing national longitudinal data from the British Millennial Cohort and objective accelerometer measurements, this study examined the impact of early childhood physical activity on adolescent emotional and peer problems. Findings revealed that MVPA at age 7 significantly predicted fewer emotional and peer relationship problems at ages 11 and 14. In contrast, LPA showed no consistent association with SB. The protective effect of MVPA was intensity-specific and enduring across ages, indicating that early physical activity may influence mental health trajectories by fostering emotional regulation and enhancing social interaction and belonging.

This study provides valuable empirical support for incorporating MVPA into mental health promotion strategies for children. Leveraging a nationally representative sample and objective physical activity data, the findings suggest that increasing daily MVPA levels during the prepubertal period, when the incidence of psychological problems is high, holds important preventive and interventional value. Future research should further explore the regulatory mechanisms underlying the relationship between exercise, social structure, sex, socioeconomic status, and child and adolescent mental health. Such investigations would enrich the theoretical foundation and practical implementation of mental health interventions targeting this population.
